# Virtual Screening of Natural Products against Type II Transmembrane Serine Protease (TMPRSS2), the Priming Agent of Coronavirus 2 (SARS-CoV-2)

**DOI:** 10.3390/molecules25102271

**Published:** 2020-05-12

**Authors:** Noor Rahman, Zarrin Basharat, Muhammad Yousuf, Giuseppe Castaldo, Luca Rastrelli, Haroon Khan

**Affiliations:** 1 H.E.J. Research Institute of Chemistry, International Center for Chemical and Biological Sciences, University of Karachi, Karachi 75270, Pakistan; noorbiochemist@gmail.com (N.R.); planck56@gmail.com (M.Y.); 2 Jamil-ur-Rahman Center for Genome Research, PCMD, ICCBS, University of Karachi, Karachi 75270, Pakistan; zarrin.iiui@gmail.com; 3 NUTRIKETO_LAB Unisa-“San Giuseppe Moscati” National Hospital (AORN), Contrada Amoretta, 83100 Avellino (AV), Italy; giuseppecastaldo@yahoo.it; 4 Dipartimento di Farmacia, University of Salerno. Via Giovanni Paolo II, 84084 Fisciano (SA), Italy; 5 Department of Pharmacy, Abdul Wali Khan University, Mardan 23200, Pakistan

**Keywords:** coronavirus, serine protease, natural product, drug design, docking

## Abstract

Severe acute respiratory syndrome coronavirus 2 (SARS-CoV-2) has caused about 2 million infections and is responsible for more than 100,000 deaths worldwide. To date, there is no specific drug registered to combat the disease it causes, named coronavirus disease 2019 (COVID-19). In the current study, we used an in silico approach to screen natural compounds to find potent inhibitors of the host enzyme transmembrane protease serine 2 (TMPRSS2). This enzyme facilitates viral particle entry into host cells, and its inhibition blocks virus fusion with angiotensin-converting enzyme 2 (ACE2). This, in turn, restricts SARS-CoV-2 pathogenesis. A three-dimensional structure of TMPRSS2 was built using SWISS-MODEL and validated by RAMPAGE. The natural compounds library Natural Product Activity and Species Source (NPASS), containing 30,927 compounds, was screened against the target protein. Two techniques were used in the Molecular Operating Environment (MOE) for this purpose, i.e., a ligand-based pharmacophore approach and a molecular docking-based screening. In total, 2140 compounds with pharmacophoric features were retained using the first approach. Using the second approach, 85 compounds with molecular docking comparable to or greater than that of the standard inhibitor (camostat mesylate) were identified. The top 12 compounds with the most favorable structural features were studied for physicochemical and ADMET (absorption, distribution, metabolism, excretion, toxicity) properties. The low-molecular-weight compound NPC306344 showed significant interaction with the active site residues of TMPRSS2, with a binding energy score of −14.69. Further in vitro and in vivo validation is needed to study and develop an anti-COVID-19 drug based on the structures of the most promising compounds identified in this study.

## 1. Introduction

Coronaviruses (CoV) belong to the family coronaviridae and are considered to be the largest RNA viruses, with genomes ranging from 27 to 32 kb [1]. They have been known to exist across the timeframe of history and cause upper and lower respiratory tract infections [2]. These viruses are considered to be enzootic (limited to their natural animal host) but somehow have managed to breach the animal–human species barrier, manifesting themselves as virulent human viruses [3,4].

Emerging viruses from this family have posed serious threats to mankind, including severe acute respiratory syndrome (SARS) and Middle East respiratory syndrome (MERS), both of which had pandemic effects resulting into 8096 and 2229 confirmed cases with a mortality rate of 9.6% and 35.5%, respectively [5]. In the same context, the recent outbreak of SARS-CoV 2 (the causative agent of coronavirus disease 2019 (COVID-19)) in December 2019 has inflicted 204 nations, with a total of 896,450 confirmed cases and 45,526 deaths up to 2 April 2020.

The sequence analysis of SARS-CoV 2 receptor-binding domain (RBD) that makes contact with angiotensin-converting enzyme 2(ACE-2) [6] revealed that most amino acids essential for SARS-S (SARS spike protein) binding are conserved in SARS-2-S and have been validated to use similar host cell receptors [7]. From a structural perspective, the genome of coronaviruses encodes four structural proteins: E (envelope protein, the smallest in size, expressed in abundance inside infected cells during the replication cycle but only partially incorporated into the virion envelope) [8], M (matrix glycoprotein, responsible for the assembly of virions [1,9] and interacting with *S* proteins, promoting their retention in the endoplasmic reticulum (ER)–Golgi intermediate for their incorporation into new virions) [10], N (nucleocapsid protein, the only protein capable of binding to the virus RNA genome, synthesizing nucleocapsid) [11], S (spike protein, mediating the attachment of the virus to host cell receptors) [12,13,14]. The S proteins aforementioned are composed of two subunits: S1, the *N*-terminus of which functions as a receptor binding region, and S2, which serves to promote fusion activity via its *C*-terminus [15]. Unraveling S protein activation is therefore key to understanding human CoV (HCoV) tropism, ecology, and pathogenesis. Like other class I viral fusion proteins, the human coronavirus spikes require proteolytic priming to be activated [16]. Notably, the majority of pathogenic HCoVs exit the producer cells with unprimed S proteins [17,18] and thus rely on target-cell proteases for activation. Therefore, HCoV cell entry factors on target cells include virus-binding agents (cell receptors) and virus protein-cleaving agents (cell proteases).

SARS-CoVs bind naturally to the ectopeptidase receptor ACE-2 with very high affinity [19], but it has been noted that the binding of *S* protein takes place distant from the ACE-2 enzyme pocket [6], indicating that ACE-2 is not a direct *S*-activating protease. Several proteases are available to serve as cofactors for viral entrance, such as cathepsin L, thermolysins, plasmins, and trypsin [20,21,22], but these proteases are mostly soluble and cannot be retained in the vicinity of ACE-2 receptors. Hence, the question regarding location of the activating protease and timing of S protein binding to it is relevant because endoproteolytic cleavage only takes place after ACE-2 engagement. Indeed, if occurring prior to engagement, binding with such proteases might cleave and inactivate the viral spikes [21,22]. Taking into account the sequence of *S* protein binding to ACE-2 and subsequent proteolytic cleavage, it would be relevant to state that the protease might be anchored in the cell membrane near ACE-2 receptors. Among the candidates for the membrane-anchored virus activating protease are the type II transmembrane serine proteases (TTSPs), expressed in the epithelial cell lining of the nose, trachea, and distal airways [23].

According to a study [7], S protein priming by cellular proteases, which entails S protein cleavage at the S1/S2 and S2′ sites (same in SARS-S and SARS-2-S), allowing the fusion of viral and cellular membranes, is a process driven by the S2 subunit engaging ACE-2 as the entry receptor and employing the cellular serine protease RSS2 [24,25,26]. It has also been mentioned that though the coronavirus strain may use cathepsin L/B or TMPRSS2 for proteolytic priming, only TMPRSS2 is essential for viral spread and pathogenicity, whereas cathepsin B/L activity is dispensable [26,27,28].

Considering the vital role played by TMPRSS2 in the priming of viral spike proteins, this protease can be used as a potential target to inhibit virus entry into host cells. This protein also binds the hemagglutinin protein of influenza and, therefore, is a potential drug target also the flu virus, besides coronavirus [29]. It is also expressed in prostate cancer and tumor metastasis [30]. TMPRSS2 expression and variants served as COVID-19 modulators in Italian patients [31]. Being part of the host cells, these proteases are also not prone to progressive mutations, which mostly occur in viral protein targets. In this study, we have identified potential natural product candidates which can inhibit these proteases efficiently.

## 2. Results

**Figure 1:**
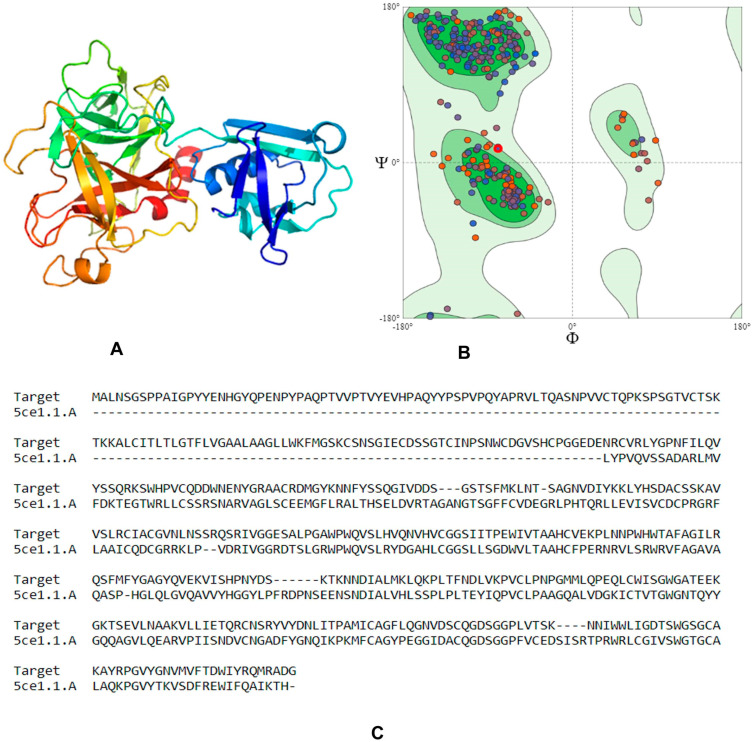
(**A**) Three-dimensional structure of the modelled serine protease transmembrane protease serine 2 (TMPRSS2), (**B**) Ramachandran plot validation of the modelled 3D structure, (**C**) alignment of the target serine protease TMPRSS2 and the template serine protease hepsin (PDB ID: 5CE1).

In the current study, we used computational biology to screen and dock a library of natural compounds to inhibit human TMPRSS2, which facilitates the entry of SARS-CoV-19 onto host cells. The three-dimensional structure of TMPRSS2 was built using the online server SWISS-MODEL, as shown in Figure 1A. We validated the results of the model obtained with SWISS-MODEL and cross-checked in RAMPAGE, observing 319 (92.7%) residues in the favored region, 23 (6.7%) residues in the allowed region, and 2 (0.6%) residues in the outlier region, which indicated the correct geometry and three-dimensional arrangement of the model, as shown in Figure 1B. Alignment of the template (PDB ID: 5CE1) and the target protein is shown in Figure 1C. 

The validated structure of human serine protease 2 was prepared for molecular docking analysis using the Molecular Operating Environment (MOE) software. The protein was 3D protonated, and energy was minimized by using energy minimization in the compute option in the MOE software. After energy minimization, the binding pocket of the protein was predicted by using the MOE site finder option to select the active site residues in the binding pocket in the three-dimensional atomic coordinates of the protein. The predicted active site residues of TMPRSS2 are Asn146, Arg147, Cys148, Val149, Arg150, Leu151, Asp187, Met188, Tyr190, Ile221, Tyr222, Lys223, Asn368, Pro369, Gly370, Met371, Lys449, Asn450, Ile452, and Trp454. 

Natural Product Activity and Species Source (NPASS) is a freely accessible database containing 30,927 natural compounds classified into 18 different superclasses, which include alkaloids and derivatives, lipids and lipid-like molecules, benzenoids, lignans, neolignans and associated compounds, and so on [32]. These natural products are found in the kingdom or super-kingdom of bacteria (6.7%), fungi (7.9%), metazoan (9.4%), and viridiplantae (67.8%) from 6814 genera. In silico screening of large databases is a cost-effective and time-saving approach towards drug discovery. In the present study, we used two approaches for compound screening, i.e., a pharmacophore-based approach and a molecular docking score-based approach. For the first compound screening approach, we selected 10 pharmacophoric features of the known inhibitor of serine protease 2 camostat mesylate, which includes anionic and cationic atoms, an H-bond donor and acceptor, an aromatic center, a Pi ring center, and a hydrophobic centroid. Based on these features of the known inhibitor, we got 2140 compounds out 30,927 in the result file. In the second compound screening approach, these 2140 compounds were docked against TMPRSS2 for the evaluation of potent inhibitors. 

The molecular docking analysis revealed 85 compounds with a docking score comparable or lower than that of the standard inhibitor camostat mesylate, an FDA-approved drug. Compounds with the lowest docking score are considered to be the most potent inhibitors. 

**Figure 2:**
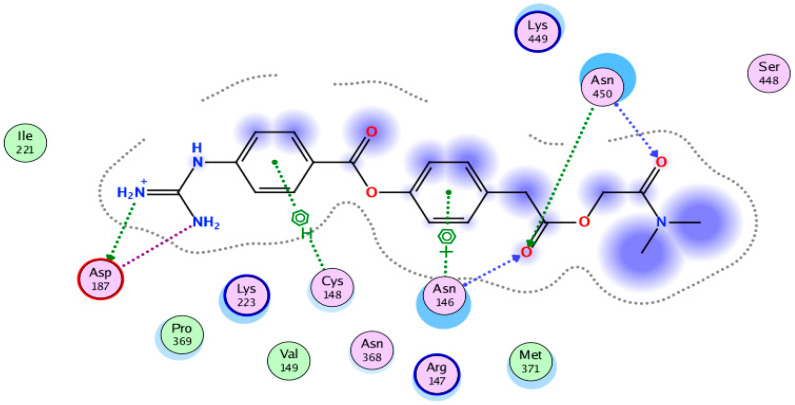
Two-dimensional interactions of camostat mesylate (standard inhibitor) with the active site residues (Asp187, Asn346, Cys348, and Asn450) of human serine protease.

**Figure 3:**
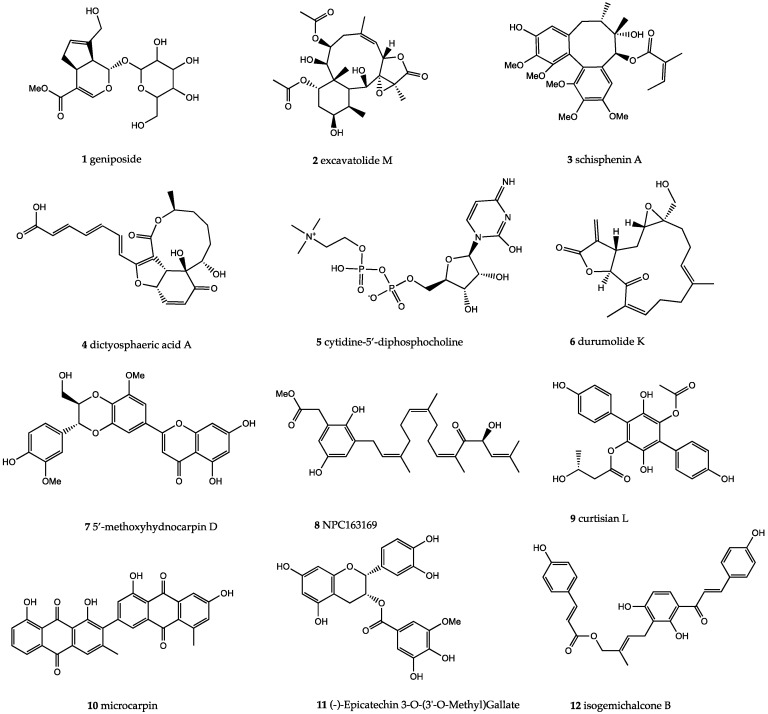
Molecular structures of the top 12 natural compounds (compounds **1**–**12**) with regard to their docking scores, potential inhibitors of TMPRSS2.

It is interesting to mention that camostat is approved by the Japanese FDA for the treatment of chronic pancreatitis and postoperative and reflux esophagitis. It could be considered for off-label treatment of SARS-CoV-2-infected patients. The standard inhibitor of TMPRSS2, i.e., camostat, interacted with four key residues of the protease active site by forming seven hydrogen bonds, as shown in Figure 2. Asparagine 146 forms an arene cation and backbone acceptor H-bonds, while Cys148 forms an arene-H bond with the benzene ring of the ligand. Asparagine 450 forms two hydrogen bonds with the anhydrous carbonyl oxygen, with sidechain acceptor and backbone acceptor. Aspartic acid 187 forms an acidic hydrogen bond with the primary amine and a sidechain donor hydrogen bond with the secondary amine of the ligand. We selected the top-ranked drug-like compounds (12 compounds, Figure 3) shown in Table 1, with a docking score equal to −13 or lower, in the attempt to identify active natural compounds for drug development. 

**Figure 4:**
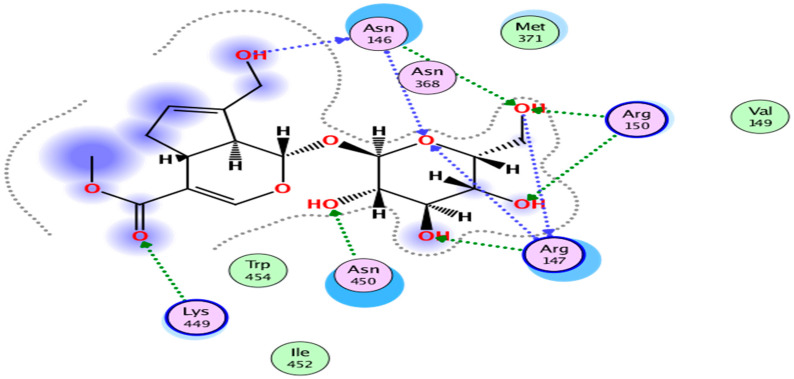
Two-dimensional interactions of NPC306344 (compound **1**) with the active site residues of human TMPRSS2. Binding site residues are Asn146, Arg147, Arg150, Lys449, and Asn450.

Among these drug-like compounds, compounds **1** (NPC306344) showed the highest docking score of −14.69. All the selected drug-like compounds showed interaction with TMPRSS2, with a docking score (<−13) better than the docking score (−11.06) of the standard inhibitor Compound **1** forms 10 hydrogen bonds with the active site residues of the receptor protein, as shown in Figure 4. Among these H-bonds, six residues (Asn146, Arg147, Arg150, Lys449, and Asn450) are sidechain acceptor, and two residues (Asn146 and Arg147) are both backbone acceptor and donor. This compound has the IUPAC name methyl (1*S*,4a*S*,7a*S*)-7-(hydroxymethyl)-1-[3,4,5-trihydroxy-6-(hydroxymethyl)oxan-2-yl]oxy-1,4a,5,7a-tetrahydrocyclopenta[c]pyran-4-carboxylate and the common name geniposide and is one of the major iridoid glycosides of gardenia fruit. It was previously shown to inhibit 5-lipoxygenase [33] and the tumor-promoting factor P-glycoprotein [34] and have anti-angiogenic activity [35] and potential antiasthma properties [36]. Additionally, geniposide was shown recently to protect against sepsis-induced myocardial dysfunction by activating AMPKα to suppress myocardial reactive oxygen species (ROS) accumulation [37]. It is present in nearly 40 species belonging to various families, especially the Rubiaceae, among which the most representative are *Tinospora capillipes* (native to China), *Paederia scandens* (native to China), *Cornus officinalis* (native to China, Japan, Korea), *Eucommia ulmoides* (native to China), *Lantana camara* (weed native to Mexico, Central America, the Caribbean, and tropical South America), *Paratinospora sagittata* (present in southern Taiwanese and mainland Chinese disjunction), *Artemisia capillaries* (Chinese medicinal herb), *Plantago asiatica* (native to China, Japan, Korea), *Rehmannia glutinosa* (native to China), *Plantago depressa* (native to China, Korea, Himalayas), and *Gardenia jasminoides* (native to Vietnam, Southern China, Korea, Taiwan, Japan, Myanmar, India, and Bangladesh). ADMET indicators suggested that this compound does not have gastrointestinal absorption capability, nor can it cross the blood–brain barrier. Regarding its metabolic role, it was found to be a substrate of CYP450 3A4 enzyme and an inhibitor of OATP1B1. It possesses estrogen and androgen receptor, as well as aromatase binding properties. Eye irritation, Ames toxicity, and carcinogenicity were null, but toxicity for honey bee and fish was high. Acute oral toxicity was 3.466 kg/mol, plasma protein binding was around 50%, and water solubility appeared low. The LD_50_ (lethal dose, 50%) for rat acute toxicity was 2.79 mol/kg, the pIGC50 (Prediction of the Toxicity) for *Tetrahymena pyriformis* was 0.2 μg/L, and the pLC50 (Predicted Toxicity Values) for fish was 1.09 mg/L. It was predicted to be localized in mitochondria, and its biodegradability was negligible or low.

**Figure 5:**
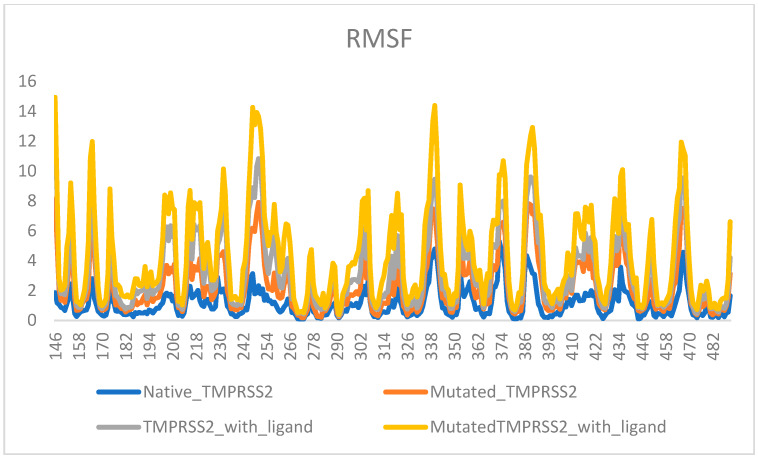
Root-mean-square fluctuation of main-chain atom coordinates of native, mutated, and ligand-bound and unbound forms of TMPRSS2, from alpha carbon backbone.

Asselta et al. [31] suggested that polymorphisms are responsible for enhanced expression of TMPRSS2 in the Italian population, and hence, increased mortality. A substitution mutation at position 160, i.e., V160M was probed from a docking perspective to see if the drug would bind differently in individuals carrying this mutation. The binding energy values of the top 12 ligands were studied for both native and mutant proteins. The values for binding to the mutant protein were different from those of binding to the native protein (with energy S value difference of 1.2). A root-mean-square fluctuation of the main-chain atom coordinates from alpha carbon traces of the native TMPRSS2, its V160M mutant, and the ligand-bound native TMPRSS2 and mutant TMPRSS2 structures was also observed (Figure 5). The binding site residues were also altered significantly in the mutant. Other top-ranked drug-like compounds, potential inhibitor of TMPRSS2 [32], were the marine natural product excavatolide M (compound **2**), a briarane-type diterpene, the cembranolide durumolide K (compound **6**), predicted as toxic, the dibenzylcyclooctadiene lignan schisphenin A (compound **3**) from *Shisandra sphenanthera*, the fungal decalactone dictyosphaeric acid A, obtained from the green alga *Dictyosphaeria versluyii*, with antibacterial activity against methicillin-resistant *Staphylococcus aureus* (MRSA), vancomycin-resistant *Enterococcus faecium*, and *Candida albicans* [38]. (compound **4**), the endogenous cytidine (5′)-diphosphocholine, known as citicoline, (compound **5**), with beneficial effects in transient global and focal cerebral ischemia [39], 5-methoxyhydnocarpin (compound **7**), a *Berberis* species flavonolignan similar to silymarin and reported as a strong inhibitor of the NorA pump (an endogenous efflux transporter of *S. aureus* in the plasma membrane [40]), compound **8** from the free-floating algae known as *Sargassum*, the *p*-terphenyl antioxidant curtisian L (compound **9**) from the wild mushroom *Paxillus*, microcarpin (compound **10**), a bianthraquinone from *Asphodelus microcarpus*, the green tea polyphenol (-)-epicatechin 3-*O*-(3′-*O*-methyl) gallate (EGCG3”Me) (compound **11**), and the aromatase inhibitor isogemichalcone B (compound **12**), mainly from *Artocarpus* and *Broussonetia* genera. Among the remaining 73 compounds with a docking score comparable to that of the standard drug inhibitor, it is worth mentioning the active principles of popular herbs used in Ayurvedic traditional medicine and components of multi-ingredient food supplements formulations, i.e., fuscaxanthone A from *Garcinia* spp. (**29**, docking score: −12.35), orthosiphonone D (**44**, docking score: −12.00) from Java tea (*Orthosiphon stamineus*), 7-hydroxy-14-deoxywithanolide U (**77**, docking score: −11.12) from *Withania somnifera*, commonly known as ashwagandha, a plant of immense medicinal properties belonging to the family Solanaceae [41], and 6*S*,9*R*-roseoside (**78**, docking score: −11.12) from *Ocimum basilicum and Ocimum sanctum* [42].

## 3. Discussion

The current study aimed to identify potential candidate molecules with the ability to inhibit SARS-CoV 2 by acting on the TMPRSS2 enzyme, responsible for the priming of the S proteins found on the surface of the virus [27,43]. These spike proteins of coronaviruses facilitate viral entry into the target cells. The S glycoprotein is composed of S1 and S2 subunits. The S1 subunit contains a signal peptide, followed by an *N*-terminal domain (NTD) and an RBD, while the S2 subunit contains a conserved fusion peptide, heptad repeats 1 and 2, a transmembrane domain, and a cytoplasmic domain [14].

It has been observed in previous studies that most human and animal cell lines, including Vero and MDCKII, are equally susceptible to virus entry driven by SARS-S and SARS-2-S, suggesting similarity in the choice of receptors by the two coronaviruses [7] Similarly, it has also been evident from sequence analysis that SARS-2-S RBM, responsible for making contact with ACE2, tends to harbor the same kind of amino acids present in SARS-S, indicating the use of ACE-2 for cellular entry [44].

Studies have also proven that ACE-2 is not a direct *S*-activating protease, as it binds through a region distant from the ACE-2 enzyme pocket (14). Hence, the protease responsible for S protein activation should be available in the vicinity of ACE-2 and be anchored in the membrane [25]. TMPRSS2, a serine protease family, is known for its longer, potentially palmitoylated 84-residue cytoplasmic tail, which may position it into lipid rafts of the plasma membrane [45]. In this context, endosomal cathepsin L (a cysteine protease) is known as an *S*-activating enzyme, capable of cleaving the *S* protein to promote viral–cellular membrane fusion [15,28]. However, only TMPRSS2 activity is essential for viral spread and pathogenesis in the infected host, whereas cathepsin B/L is dispensable [19,24,26,27,28]. 

Several serine protease inhibitors have been designed and are available in the market, including camostat mesylate (used as a standard inhibitor in this study), nafamostat mesylate, bromohexine hydrochloride, and the protein inhibitors PAI-1 or HAI-2 [29]. In this study, we focused on the inhibition of TMPRSS2, a host serine protease which is less prone to mutations over time compared to viral proteins, using natural products. For this purpose, we adapted computational analysis to screen and dock a library of natural compounds considered as potential inhibitors of TMPRSS2. Camostat mesylate (a Japanese approved drug, trade name Fiopin) was taken as a standard drug to compare docking scores and physicochemical parameters. The standard drug is known to inhibit syncytium formation [27,46]. An online SWISS-MODELL homology structure of TMPRSS2 was generated and tested by using the RAMPAGE tool, showing 92.7% residues in the favored region and 6.7% in the allowed ones, thus indicating correct geometry and 3D arrangement. The active site was deduced using the MOE software. 

NPASS, a freely accessible database containing 30,927 compounds, was used to mine the potent inhibitors. As a result, 2140 compounds were identified as potent candidates, after the first phase of physicochemical analysis. The compounds obtained were docked against TMPRSS2 in the second phase of the analysis. The second phase identified 85 compounds with binding energies comparable to or lower than that of the standard inhibitor camostat mesylate (−11.06). Among these compounds, we focused on the compounds with the lowest docking scores, also following the Lipinski rule of five shown in Table 1. 

## 4. Methods

### 4.1. Homology Modeling

The amino acid sequence of human transmembrane protease serine 2 (Uniprot accession no: O15393) isoform-2 is 492 amino acids long and was chosen for analysis. It was retrieved in FASTA format and uploaded to the online server SWISS-MODEL (https://swissmodel.expasy.org/) to build a homology model of the target protein. The three-dimensional modelled structure was validated by uploading on the RAMPAGE server (http://mordred.bioc.cam.ac.uk/~rapper/rampage.php).

### 4.2. Compounds Library Screening

We screened the Natural Product Activity and Species Source database (NPASS, available at http://bidd2.nus.edu.sg/NPASS) which contains 30,927 compounds [32]. Pharmacophore- and molecular docking-based screening were performed, using the MOE software. In pharmacophore-based screening, we selected the pharmacophoric features of the standard serine protease inhibitor camostat mesylate, a trypsin-like protease inhibitor, for compound mining. The pharmacophore-based screened library of compounds was further docked with the target protein to identify lead compounds with the best docking scores. 

### 4.3. Molecular Docking and Downstream Analysis

For molecular docking analysis, the three-dimensional structure of human TMPRSS2 in .pdb format was opened in MOE software (Chemical Computing Group, Montreal, Quebec, Canada). Then, 3D protonation and energy minimization were done with compute option, till a gradient of 0.05 was reached. Polar hydrogens were added, and site finder was used to predict the active site residues in the binding pockets of the protein. For molecular docking, we selected the .mdb file of the library and used the following parameters: placement: triangle matcher, Refinement: Rigid receptor, poses: 1, and Rescoring 1 and 2: London dG. The docked compounds were ranked based on their docking scores [29]. The compounds with the best docking scores were further evaluated according to the Lipinski’s rule of five for ligand properties in MOE software. ADMET property analysis was done using admetSAR2.0 (http://lmmd.ecust.edu.cn/admetsar2/). Based on a report that TMPRSS2 mutation makes some individuals more susceptible to COVID-19, a reported mutation V160M suggestive of changed disease susceptibility [31] was studied with respect to its docking conformation in the presence of the top compound. The protein residue was modified in PyMoL using the mutagenesis function. The mutated protein was docked, and structural binding of the apo- and holo-forms of native and mutated TMPRSS2 proteins was studied. Visualization was done in PyMol and MOE.

## 5. Conclusions

Protein–ligand interaction is a potent approach to mine drugs. Human judgment regarding these interactions has been facilitated by computational approaches. This study made use of a computational approach to sift out interactions that are beneficial for and exclusive to TMPRSS2 binding and, hence, curb SARS-CoV-2 priming. Compounds showing interactions with low scores or unfavorable parameters were excluded, while those able to establish favorable interactions were retained. The docking score of compound **1** was found to be −14.69, indicating this compound as the best drug candidate, among those with promising features, for drug development. Its binding site and energy value were altered when it was docked with the TMPRSS2 V160M mutant. This information adds an important and flexible dimension to natural-drug mining against COVID-19 and has led to some concrete predictions, which would hopefully be confirmed experimentally. urther in vitro and in vivo testing of the compounds identified in this study is necessary, prior to their input in the clinical trial pipeline.

## Figures and Tables

**Table 1:** Docking scores of the top compounds with the lowest binding energies. Their molecular properties are shown along with toxicity. Compound **2** and **6** were predicted as toxic.

S.no.	Compound ID	Docking Score	Toxicity	M.Wt (g/mol)	H-Bond Donor	H-bond Acceptor	LogP	LogS
**1**	NPC306344	−14.69	No	388.37	5	9	−1.45	−0.72
**2**	NPC473877	−14.38	Yes epoxide	482.53	3	7	0.38	−3.04
**3**	NPC470916	−14.27	No	516.59	2	8	4.16	−5.62
**4**	NPC66108	−14.02	No	416.63	3	7	1.8	−3.78
**5**	NPC328914	−13.96	No	488.33	5	12	−3.94	−0.53
**6**	NPC476270	−13.92	Yes epoxide	346.42	1	4	2.67	−3.75
**7**	NPC84324	−13.59	No	494.45	4	9	3.41	−5.43
**8**	NPC163169	−13.55	No	484.63	3	5	7.03	−7.00
**9**	NPC155015	−13.38	No	454.43	5	7	3.84	−5.22
**10**	NPC19631	−13.31	No	506.47	4	8	6.47	−8.21
**11**	NPC53889	−13.10	No	456.40	6	9	2.03	−4.05
**12**	NPC19622	−13.07	No	486.52	4	6	5.47	−6.55
**13**	Camostat mesylate(standard)	−11.06	No	399.43	3	3	0.23	−2.83
